# Lung cancer mortality in Montenegro, 1990 to 2015

**DOI:** 10.3325/cmj.2019.60.26

**Published:** 2019-02

**Authors:** Mirjana Nedović-Vuković, Dragan Laušević, Agima Ljaljević, Mileta Golubović, Goran Trajković

**Affiliations:** 1 Institute of Public Health of Montenegro, Podgorica, Montenegro; 2 Faculty of Medicine, University of Montenegro, Podgorica, Montenegro; 3 Institute of Medical Statistics and Informatics, Faculty of Medicine University of Belgrade, Belgrade, Serbia

## Abstract

**Aim:**

To analyze the trend of lung cancer mortality in Montenegro from 1990 to 2015.

**Methods:**

Data on lung cancer mortality were collected from death certificates obtained from the Statistical Office of Montenegro for the period 1990-2009 and the Institute for Public Health for the period 2010-2015. Population data were obtained from the Statistical Office of Montenegro. Rates were age-standardized to the World Standard Population, and mortality trends were analyzed with the joinpoint regression.

**Results:**

In 2015, lung cancer accounted for 5.44% of all deaths and 22.92% of all cancer deaths. It was the leading cause of all cancer deaths and the third-leading cause of all deaths. A joinpoint was observed in 2004 in women and in the entire population, and in 2005 in men. The overall mortality rates increased from 1990 to 2004 by an average of 3.91% per year and decreased from 2004 to 2015 by an average of 1.95%; which in the entire observed period resulted in an average increase of 1.3% per year. A particularly strong growth rate was observed in women, even 7.14% in the period from 1990 to 2004.

**Conclusion:**

The observed increase in lung cancer mortality warrants improved tobacco control.

Lung cancer is the main cause of all cancer deaths among men, and it has replaced breast cancer as the main cause of all cancer deaths among women in more developed countries (1). In 2012, lung cancer caused 24% of cancer deaths in men and 14% in women worldwide (1,2). In the same year there were also approximately 1.6 million lung cancer-related deaths, and by 2035 this number is expected to increase by 86% (1-3). The highest risk factor for lung cancer is smoking, even smoking a few cigarettes per day (4). As many as 80% of lung cancer cases in men and 50% in women are caused by smoking (5,6), making tobacco control the key strategy for lung cancer prevention (2,7,8). The process of active tobacco control in Montenegro was initiated in 2003 by establishing the National Tobacco Control Commission and developing the National Strategy for Tobacco Control (9), which derives fundamental concepts from international documents related to tobacco control in Europe (10-12). It introduced measures (13-16) and law regulations (17,18) for improving tobacco control in Montenegro (19).

In the period 1976 to 2000 an increasing trend of lung cancer mortality was observed in Montenegro, with a significant correlation between lung cancer mortality rate and tobacco products use (20). More recent trends have not been analyzed and it is not known whether the trend of lung cancer mortality changed after the changes in smoking prevalence at the beginning of the 21st century (21-23). The aim of this article is to analyze the trend of lung cancer mortality in Montenegro for the period 1990-2015 by using joinpoint regression (24,25).

## MATERIAL AND METHODS

### Data sources

The study gathered data on lung cancer mortality in Montenegro from 1990 to 2015. The primary data source were death certificates completed by physicians who determined the time and cause of death. The certificates for the period until 2009 were obtained from the Statistical Office of Montenegro and for the period after 2009 from the Institute for Public Health. Lung cancer was defined by using the International Classification of Diseases code 162 from the 9th edition, and codes C33 and C34 from the 10th edition (26). The number of death cases of lung cancer was published in the Statistical Yearbooks of the Institute of Public Health for the period 1999-2009 (27). Population data were obtained from the Statistical Office of Montenegro.

### Statistical analyses

Rates were age-standardized to the World Standard Population (28) for the estimation of overall and sex-related trends. The joinpoint regression model was used to analyze long-term trends in lung cancer mortality and detect the time points when there was a significant change in the linear time trend. In the joinpoint regression model, the dependent variable x is the year and the independent variable y is the logarithmically transformed mortality rate. The models also estimated annual percentage change and average annual percentage change of lung cancer rates. Statistical analysis was carried out with the Joinpoint software, version 4.6.0.0 (April 2018, available from the Surveillance Research Program of the US National Cancer Institute) (29). The selected method for analysis was Grid-search method. The minimum number of observations for the number of points from the end of the time series to the first joinpoint was set to 3, and the minimum number of observations between two joinpoints was set to 4. The number of joinpoints was set between 0 and 4. The permutation test was used to choose the best fit joinpoint model, with the total significance level of 0.05. The difference between men and women was determined with the parallelism test (30).

## RESULTS

In 2015, lung cancer ranked third among all death causes and accounted for 5.44% of all deaths (6.68% in men, 2.79% in women) and for 22.80% of all cancer deaths (30.41% in men, 13.23% in women). In terms of total cancer mortality, it ranked first among men and second among women.

The total number of deaths from lung cancer increased from 163 in 1990 to 297 in 2015 (133 to 222 in men, 30 to 75 in women), which equals to an increase of 82.2% (66.9% in men and 150% in women). Age-standardized rates ranged from 19.3/100 000 in 1993 to 33.8/100 000 in 2005. In men, age-standardized rates increased from 40/100 000 in 1990 to 43/100 000 in 2015, and in women from 6/100 000 to 13/100 000. Death rate ratio between men and women in 1990 was 5.85. Rates among women were rising faster and approached the rates in men. Therefore, the ratio in 2015 was 43.10% lower than in 1990 (Table 1).

**Table 1:** Deaths from lung cancer in Montenegro from 1990-2015

	Men		Women		All		Rate ratio male/female
Year	n	ASR*	n	ASR	n	ASR
1990	133	40.3	30	6.9	163	22.2	5.8
1991	124	40.2	28	7.1	152	21.6	5.7
1992	106	32.3	25	6.3	131	19.9	5.1
1993	113	33.9	31	7.8	144	19.3	4.3
1994	151	43.0	23	5.5	174	22.4	7.8
1995	153	43.1	33	7.1	186	23.4	6.1
1996	134	36.7	35	7.8	169	20.9	4.7
1997	156	41.1	37	8.3	193	23.0	5.0
1998	153	40.1	39	8.2	192	22.6	4.9
1999	192	49.5	35	7.6	227	26.6	6.5
2000	207	50.7	65	12.9	272	30.2	3.9
2001	199	48.6	57	11.5	256	28.6	4.2
2002	204	50.3	65	13.3	269	30.3	3.8
2003	224	54.7	71	15.0	295	33.2	3.6
2004	213	51.5	74	15.6	287	31.7	3.3
2005	234	55.0	77	16.1	311	33.8	3.4
2006	206	47.9	70	13.8	276	29.2	3.5
2007	232	54.6	61	11.9	293	31.3	4.6
2008	217	48.7	70	13.6	287	29.4	3.6
2009	208	46.5	65	13.4	273	28.2	3.5
2010	228	50.0	73	14.0	301	30.3	3.6
2011	250	53.3	77	14.2	327	31.9	3.7
2012	214	44.2	80	15.3	294	28.3	2.9
2013	194	40.4	70	12.3	264	25.0	3.3
2014	189	36.7	76	13.4	265	24.4	2.7
2015	222	43.8	75	13.2	297	27.04	3.3

*ASR – rate per 100 000 age-standardized to World Standard Population.

Joinpoint regression showed a joinpoint in 2004 for the entire population and for women, and in 2005 for men. In the observed period, lung cancer mortality rates significantly increased in the entire population (average annual percentage change [AAPC] = 1.3%; 95% confidence interval [CI] 0.4-2.1; *P* < 0.001) and in women (AAPC = 3.5%; 95% CI 2.1-4.9; *P* < 0.001) and not significantly in men (AAPC = 0.6%; 95% CI -0.4 to 1.6; *P* = 0.300) (Table 2).

**Table 2:** Results of joinpoint regression analysis of lung cancer mortality in Montenegro from 1990-2015

Category	Average annual percentage change	95% confidence interval	*P*
Men	0.6	-0.4-1.6	0.300
Women	3.5	2.1-4.9	<0.001
All	1.3	0.4-2.1	<0.001

**Figure 1:**
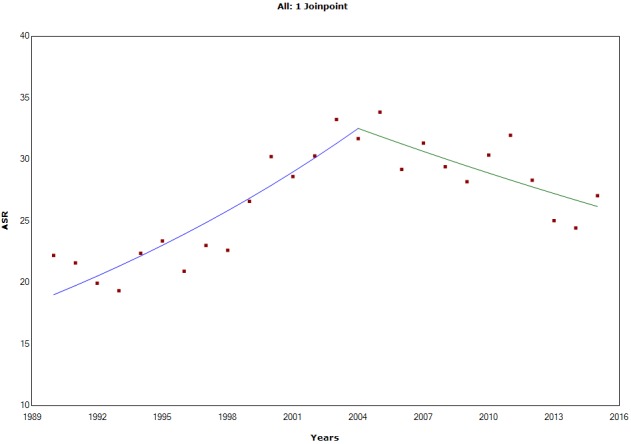
Joinpoint regression analysis of overall lung cancer mortality in Montenegro from 1990 to 2015. ASR – rate per 100 000 age-standardized to World Standard Population. Red square – observed; blue line – 1990-2004 annual percent change (APC) = 3.91*; green line – 2004-2015 APC = -1.95*. Asterisk indicates APC that is significantly different from zero at alpha = 0.05.

**Figure 2:**
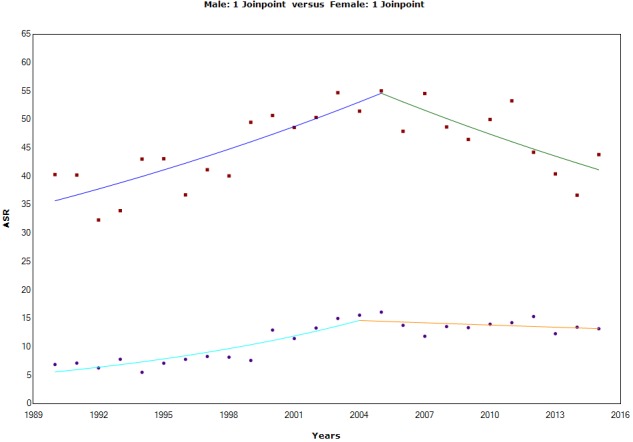
Joinpoint regression analysis mortality of lung cancer in Montenegro. Parallel pairwise comparison between sexes from 1990-2015; ASR – a rate per 100 000 age-standardized to World Standard Population. Red square – men; blue line – 1990-2005 annual percent change (APC) = 2.88*; green line – 2005-2015 APC = -2.79*; purple dot – female; light blue line – 1990-2004 APC = 7.14*; brown line – 2004-2015 APC = -0.93. Asterisk indicates APC that is significantly different from zero at alpha = 0.05; Parallelism refused, APC difference = 2.8, 95% CI 0.6-6.2, *P* < 0.001.

The overall mortality rates increased from 1990 to 2004 (annual percent change [APC] = 3.91%, 95% CI 2.7-5.1; *P* < 0.001) and decreased from 2004 to 2015 (APC = - 1.95%; 95% CI - 3.3 to -0.6; *P* < 0.001) (Figure 1). In men, the rates increased to 2005 (APC = 2.88%; 95% CI 1.6-4.1; *P* < 0.001), after which they decreased by an average 2.79% per year (95% CI -4.7 to -0.9; *P* < 0.001). In women, they were increasing faster than in men, with the rate of 7.14% in the period 1990-2004 (APC = 7.14%; 95% CI 5.1-9.2; *P* < 0.001). After 2004, the rates in women remained unchanged (*P* = 0.400). The parallelism test showed that the mortality trend line of lung cancer in men and women was not parallel (*P* < 0.001) (Figure 2).

## DISCUSSION

The joinpoint analysis showed that standardized rates of lung cancer mortality in Montenegro peaked in the early 21st century. The rates were growing in the overall period on average by 1.3% per year. The trend changed in the entire population and in women after 2004, and in men after 2005. About the same time, smoking prevalence in Montenegro decreased from 43.8% in 2000 to 32.7% in 2008 and 31% in 2012 (21-23). However, since it usually takes two to three decades for changes in smoking behavior to affect lung cancer trends (7), we cannot be certain whether these changes can be attributed to decrease in the smoking prevalence or some other factor. At the same time, it is not clear whether the lung cancer mortality trend will continue to rise, but the smoking prevalence of 36.2% in men and 34.5% in women found in 2017 (31) is still high, warranting the introduction of new laws on tobacco ban and better enforcement of the existing laws.

The estimated AAPC of lung cancer standardized mortality rate was lower than the rate of 7.7% per year observed in China 1991-2013 (32), and higher than the rate in Croatia (33). While the decreasing trend of cancer mortality in Montenegro started in the early year of 21st, in the countries of South-Eastern Europe, age-standardized lung cancer mortality rates in men started to decrease already in the period 1999-2008: by 1.2% per year in Slovenia, 0.8% per year in Croatia, and 2.5% in Malta, but they increased in Bulgaria by 2.0% and Serbia by 2.2% (34). In women, the rates decreased by 2.1% in Slovenia, 3.1% in Serbia, and 3.8 in Romania (34). European Union countries saw the peak of lung cancer mortality in men in the late 1980s, followed by a decline accompanying reduced tobacco consumption. In women, on the other hand, the mortality started growing from 1970 (35).

The variations in lung cancer mortality rates and trends between countries and between men and women within each country largely reflect differences in smoking prevalence (6,36-38). Some European countries have managed to substantially reduce smoking prevalence (39). Countries ranked high on Tobacco Control Scale 2016, such as Norway and Iceland (40), from 1980 to 2012 successfully reduced smoking prevalence by more than 50% in both men and women (41). The lack of smoking prevalence data in Montenegro prevents monitoring trends and projections of smoking prevalence reduction (42).

To facilitate prevention, as the most cost-effective long-term strategy for cancer control (43), it is important to understand lung cancer epidemiology and its causative risk factors. Determining lung cancer trends is also of crucial importance for national planning programs for lung cancer control (8,44,45).

A limitation of our study is the quality of death records, including data validity and reliability, especially since no study so far has analyzed the quality of mortality data in Montenegro. Also, different physicians were responsible for coding death causes before and after 2009 and two different classification systems, ICD-9 and ICD-10, were used. Furthermore, no studies on lung cancer causes have been conducted in Montenegro, so no precise conclusion could be made on the causes of changes registered by joinpoint regression.

In conclusion, this study observed an increase in lung cancer mortality rates during the studied period, which warrants improvement and full implementation of tobacco control as the main strategy for combating tobacco use.
